# Evolving understanding of antibody-dependent enhancement (ADE) of SARS-CoV-2

**DOI:** 10.3389/fimmu.2022.1008285

**Published:** 2022-10-18

**Authors:** Yalong Yang, Fenghua Xu

**Affiliations:** ^1^ Pharmaceutical Sciences Research Division, Department of Pharmacy, Medical Supplies Center, People’s Liberation Army (PLA) General Hospital, Beijing, China; ^2^ Medical School of Chinese People’s Liberation Army (PLA), Beijing, China

**Keywords:** SARS-CoV-2, antibody-dependent enhancement (ADE), mechanism, receptor-mediated, multiple roles of macrophages, countermeasures

## Abstract

Since immune system and internal environment *in vivo* are large and complex, the interpretation of the observed immune effect from the perspective of a single immune cell or antibody seems a little feeble. Many studies have shown that specific antibodies against “ former” viruses have a reduced ability to neutralize “new” mutant strains. However, there is no comprehensive and clear view of whether there will be Antibody-dependent enhancement (ADE). We review the latest relevant studies, hoping to explain the ADE of SARS-CoV-2 infection sometimes observed in some patients.

## Introduction

Antibody-dependent enhancement (ADE) has been observed in many coronaviruses, such as Feline infectious peritonitis (FIP) virus, SARS-CoV and Middle East Respiratory Syndrome Coronavirus (MERS-CoV) (1), which raises concerns about a possible aggravation of SARS-CoV-2 infection due to preexisting antibodies (2). Most of the studies on the mechanism of ADE of SARS-CoV-2 are related to the severity of infection. There is increasing evidence that ADE of SARS-CoV-2 does exist although rarely observed. At present, there is a lack of comprehensive and in-depth research on its specific mechanism. Results of different researches are even controversial. Different results are observed in different tissues of the body (3, 4). The results observed *in vivo* or *in vitro* also differ (5). Different results were observed even for the same cells (6). We review some recent researches on the mechanism of ADE of SARS-CoV-2, and summarize relevant viewpoints and countermeasures, as well as multiple roles of macrophages in SARS-CoV-2 infection, in order to benefit the research on ADE of SARS-CoV-2.

## The current possible mechanisms of antibody-dependent enhancement (ADE) of SARS-CoV-2

**Figure 1:**
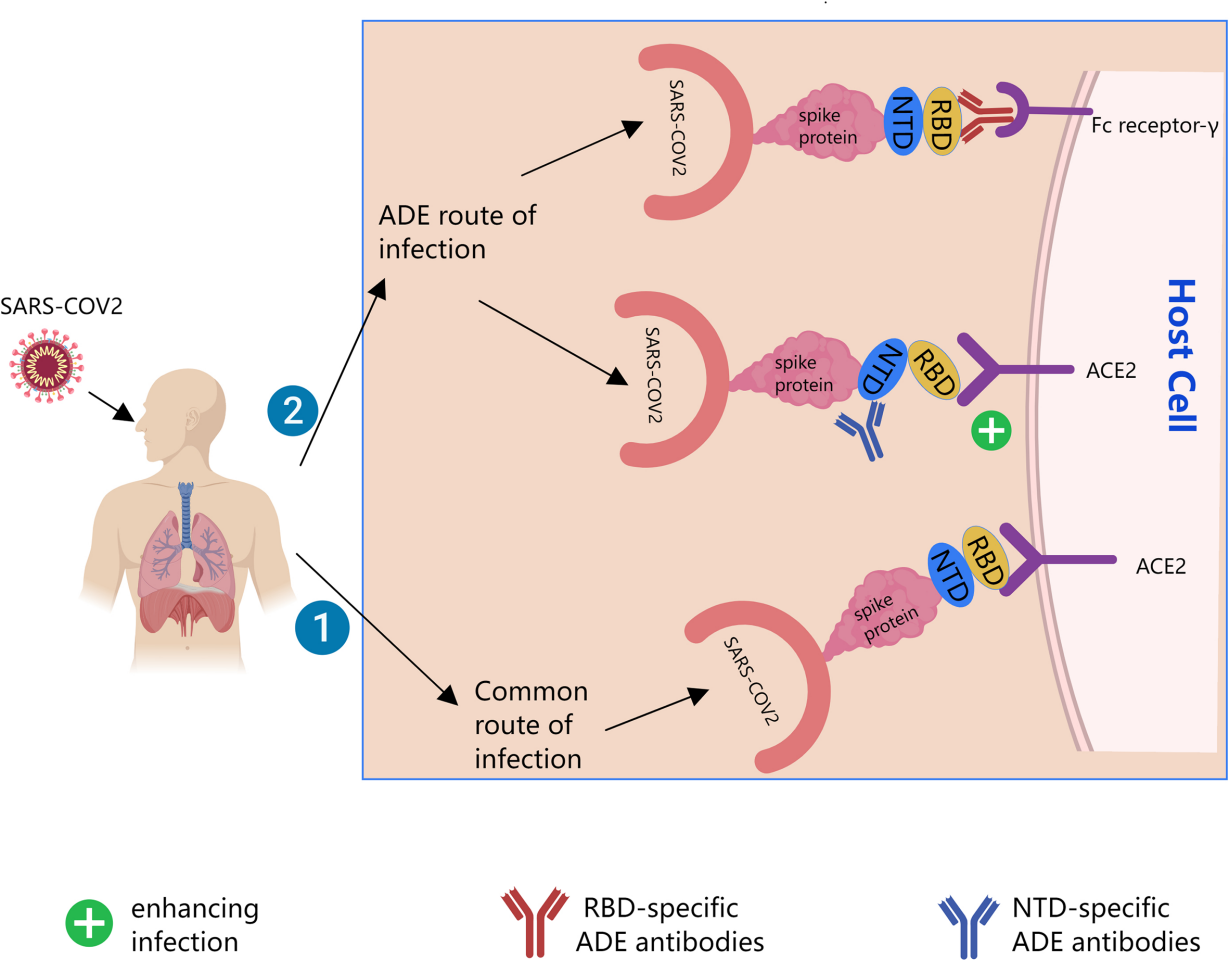
Possible mechanisms of Antibody-dependent enhancement (ADE) of SARS-CoV-2. The black line is the path direction of the infection. Pathway 1 is common pathway by which SARS-CoV-2 infect cells. Pathway 2 is possible ADE pathway by which SARS-CoV-2 infect cells. ACE2, angiotensin-converting enzyme 2; RBD, receptor binding domain; NTD, the N-terminal domain.

Several previous studies (2–8) showed that ADE of SARS-CoV-2 infection had at least two mechanisms. The Receptor binding domain (RBD)-specific ADE antibodies enhance infection depending on Fc receptors. The N-terminal domain (NTD)-specific ADE antibodies are independent of Fc receptors. Instead, they affect the binding of S protein to Angiotensin-converting enzyme 2 (ACE2) receptor by changing the conformation of S protein. Neutralizing antibodies to the RBD of SARS-CoV-2 or the NTD of SARS-CoV-2 both mediate ADE effects, as shown in **Figure 1**. And some nonspecific antibodies also mediate ADE effects.

## Potential antibodies, corresponding receptors and cells currently mediating antibody-dependent enhancement (ADE) of SARS-CoV-2

In 2020, *Wang* et al. (5) reported a monoclonal antibody (mAb)-MW05, which had SARS-CoV-2 neutralizing activity by disrupting the interaction of RBD with ACE2 receptor. ADE mediated by cross-linking of the Fc of MW05 with FcγRIIB provided the first evidence that SARS-CoV-2 monoclonal antibodies had an ADE effect *in vitro*, which could be eliminated by introducing LALA mutations into the Fc region (MW05/LALA). Their latest study in 2022 (4) reported that two neutralizing monoclonal antibodies, MW01 and MW05, could enhance the infection of FcγRIIB-expressing B cells by SARS-CoV-2 pseudovirus. This identified a novel ADE mechanism that FcγRIIB mediated the uptake of bivalent virus-antibody complexes for the SARS-CoV-2 pseudovirus *in vitro*. FcγRIIB (CD32B) is mainly expressed on myeloid cells, such as leukocyte lineages (Raji, THP-1 and K562), B cells (the only FcR on the surface of B cells), and plays an important role in the negative regulation of B cell function.

Some researchers studied the infection mechanism of ADE of SARS-CoV-2 using convalescent plasma from Coronavirus disease 2019 (COVID-19) patients and found that the ADE was mainly mediated by two types of FcγRs, FcγRIIA and FcγRIIIA (3). FcγRIIA (CD32A) is mainly expressed on the surface of neutrophils, monocytes, platelets and DC cells. It has been shown that the IgG lacking core fucosylation could initiate enhanced antibody-dependent cytotoxicity by increasing affinity for the Fc receptor FcRIIIA (9). FcγRIIIA (CD16A) is expressed on almost all leukocytes, mainly natural killer cells (NK) and macrophages.

The study of Liu et al. (7) screened some anti-spike monoclonal antibodies from patients with COVID-19. Mutational analysis revealed that all antibodies enhancing infection recognized a specific site on the NTD. Structural analysis showed that all infectivity-enhancing antibodies bound NTDs in a similar manner. And by inducing the open conformation of RBD, the binding ability of the spike protein to ACE2 and the infectivity of SARS-CoV-2 were enhanced. Not only neutralizing antibodies but also ADE-enhancing antibodies are produced during SARS-CoV-2 infection.

## Multiple roles of macrophages in SARS-CoV-2 infection

Macrophages play multiple roles in viral infection. First, they can remove the virus, infected cells or debris. Second, although they do not directly damage the lymphoid organs, they can act as a SARS-CoV-2 Trojan horse, facilitating the hiding and spreading of the virus (10–12), thereby promoting the formation of syncytia, which could target the lymphocytes for internalization and cell-in-cell (CIC) mediated death, contributing to lymphopenia (13). Third, they can enhance the recruitment of NK and Cytotoxic T lymphocyte (CTL) at the site of infection, which may kill the infected autologous tissue cells, damage the lymphoid organs. In addition, they can also recruit a variety of lymphocytes and maintain their activity.

Alveolar macrophages are sentinels for immune surveillance of foreign viruses (14). However, macrophages are diverse and are mainly divided into two categories according to their origin, alveolar macrophages (AM) and recruited monocyte-derived macrophages (MDM). Their interaction mechanisms with viruses are not exactly the same. Among them, AM (12) are polarized to M1AM and M2AM, and AM in humans seems to be biased towards M2AM. M1AM promote infection (ADE) and enhance inflammation. M2AM limit viral spread, and suppress inflammation, and enhance viral clearance. Recruited MDM are polarized into M1 and M2 (15). Some studies have found that both M1 and M2 inhibit viral infection. However, some studies have claimed that they have both pro-infection and scavenging effects. M1 destroy pathogens by producing large amounts of pro-inflammatory cytokines. M2 exhibit anti-inflammatory properties and higher phagocytic activity against pathogens. In the early stages of infection, monocyte-derived macrophages recruited to the lung clear infected cells and cellular debris (16). While tissue-resident alveolar macrophages (M1AM) promoted early infection in the lungs. Research has found that human ACE2-overexpressing or knockdown AMs don’t have a major impact on uptake of SARS-CoV-2, suggesting that ACE2 is dispensable for M1AMs to efficiently take up SARS-CoV-2. Mechanical softness could be used to reflect deformability, with greater the deformability comes greater ability to absorb extracellular particles. M1 are much softer than M2 AMs, which favors M1 AMs to efficiently take up SARS-CoV-2. On the other hand, M1 AMs have a lower endosomal pH, favoring membrane fusion and allowing the entry of viral RNA from the endosomes into the cytoplasm, where the virus achieves replication and is packaged to be released.

As the infection progresses, alveolar macrophages are largely depleted. Recruited monocyte-derived macrophages gradually surpass and replace alveolar macrophages. And Lungs have more and more activated M1 macrophages, M1 release a large number of pro-inflammatory factors, which are both beneficial to fight against SARS-CoV-2, and aggravate the inflammatory response, causing severe COVID-19 inflammation. This provides one explanation for the increasing of severe morbidity and mortality in people who are older or have underlying medical conditions, such as hypertension and diabetes (15, 17).

SARS-CoV-2 infects resident CD169^+^ macrophages in the spleen and lymph nodes *via* the ACE2 receptor, potentially leading to spleen and lymph node damage, lymphopenia (18). Compared with the normal healthy control group, the total lymphocyte counts were significantly lower in sections from viruses infected spleens, which were was mainly necrotic and apoptotic lymphocytes. This result is also consistent with the observation by Diao et al. (19) that T cell count in COVID-19 patients were significantly reduced, and that surviving T cells were functionally exhausted. There may be several reasons for this. First, the resident macrophages aggravated the infection by promoting the spread of the virus. Second, phagocytosis and clearance of macrophages at the site of infection promoted the recruitment of various cells, such as NK and CTL, which might attack infected lymphoid organs. Furthermore, SARS-CoV-2 could induce lymphocyte apoptosis by enhancing Fas signaling, and could also trigger macrophage secretion of IL-6 and promote lymphopenia.

When COVID-19 occurs, within the alveolar space, SARS-CoV-2 infects alveolar macrophages. And the infected alveolar macrophages promote chemotactic recruitment of T cells and monocytes (10). The recruited T cells produce γ-interferon, which in turn promotes the secretion of pro-inflammatory factors from alveolar macrophages and promotes further activation of T cells. A positive feedback loop exists between SARS-CoV-2-infected alveolar macrophages and T cells, causing persistent alveolar inflammatory response.

## Differences in *in vivo*/*in vitro* results of antibody-dependent enhancement (ADE) of SARS-CoV-2

Studies have pointed out that anti-recombinant native full-length S protein trimer (triSpike) antibody could mediate SARS-CoV entry into B cells through FcγRII *in vitro (* 20). And the recombinant trimeric S protein was also able to elicit potent protective immune response *in vivo*. Li et al (8). isolated neutralizing antibodies (NAbs) from individuals with a history of acute or convalescent SARS-CoV-2 or SARS-CoV-1 infection. These antibodies raised against two different crucial domain of S glycoprotein, RBD and NTD, had neutralizing activity, protecting against SARS-CoV-2 infection. They also isolated some non-neutralizing antibodies targeting RBD and NTD, which could enhance viral infection mediated by Fc receptors. Five non-neutralizing NTD antibodies could enhance FcγR-independent infection *in vitro*. While, two of them that enhanced infection *in vitro* inhibited SARS-CoV-2 replication in both monkeys and mice. These studies suggest that although antibodies can enhance infection *in vitro*, they do not necessarily predict enhanced infection *in vivo*. The possible reason is that non-neutralized NTD Abs can enhance S binding to ACE2 to enhance infection. As the environment is single and few influencing factors *in vitro*, the enhancement of infection at the level of individual cells is the main manifestations. However, *in vivo*, non-neutralized NTD Abs not only mediate ADE, but may also mediate Antibody-dependent cell-medicated cytotoxicity (ADCC), Complement dependent cytotoxicity (CDC), and promote the phagocytosis and clearance of dendritic cell (DC)and macrophages. After the virus enters the body, the immune system is activated. The result at this time depends on a net balance of infection enhancement and virus clearance level. Therefore, ADE in SARS-CoV-2 infection and vaccination may be less easily observed. Only in severe infection or immunocompromised conditions, it may be observed.

## Conclusion

Theoretically, SARS-CoV-2 has the possibility of ADE. Although there is clear evidence that antibodies against the original viruses have reduced neutralization capacity against mutant strains, there is lack of robust data for a clear ADE effect. Macrophages, which are well studied in ADE, are also found to have multiple roles.

Macrophages are scavengers and Trojan horse in the early, middle and late stages of SARS-CoV-2 infection, which do not directly contribute to ADE, but can be mediated by antibodies to facilitate viral transmission and enhance infection. Macrophages from different sources promote ADE in different stages, but also have a clearing function. In the inflammatory environment of infection with SARS-CoV-2, immune cells such as macrophages, NK, T, and B, non-immune cells such as alveolar cells and epithelial cells, various antibodies and various cytokines affect and interact with each other. The extensive expression of receptors such as pattern recognition receptor, Fc receptors, and the crosstalk between them form a complex dynamic immune network. This network affects the balance between virus clearance, ADE effect, and the extent of the inflammatory response, which determines the treatment outcome of infected patients.

Some studies have proposed some possible ways to reduce the risk of ADE of SARS-CoV-2. For example, the use of a biomimetic shell avoids the risk of ADE of SARS-CoV-2. Biodegradable calcium phosphate-encapsulated viral particles evade recognition by pre-existing antibodies extracellularly, thereby eliminating the ADE of viral infection (21). Two leucine-alanine substitutions (LALA mutations) were introduced at residues 234 and 235 of the Fc part to reduce Fc-mediated ADE (5, 22). Fucosylated anti-SARS-CoV-2 antibodies may reduce affinity for FcRIIIa receptors, thereby attenuating ADE (9). Novel activators are used to induce or deliver potent neutralizing antibodies. When infecting a mutant virus, it promotes the presentation of specific antigens against the mutant virus, restarts the immune system, and produces high-quality specific neutralizing antibodies.

In addition, ADE is not easy to occur, which may require many conditions. Such as the subtype of the antibody, the quality, specificity, titer, affinity of the antibody, and antibodies against “former” viruses have reduced neutralization capacity against mutant strains etc (23). In addition, race, genetic diversity, age, gender, vaccination/infection history, underlying health conditions, etc. may all have an impact. At present, vaccination is the best way to prevent SARS-CoV-2 infection, which can reduce the severe case fatality rate. But special populations (elders, people with underlying diseases or chronic diseases, etc.) need to be vigilant against ADE of SARS-CoV-2.

## Author contributions

YY completed writing of the paper. FX designed the paper and guided writing. All authors contributed to the article and approved the submitted version.

## Funding

This work is supported by the National Natural Science Foundations of China [82171814]. The funders have no roles in study design, data collection and analysis, decision to publish, or preparation of the manuscript.

## Conflict of interest

The authors declare that the research was conducted in the absence of any commercial or financial relationships that could be construed as a potential conflict of interest.

## Publisher’s note

All claims expressed in this article are solely those of the authors and do not necessarily represent those of their affiliated organizations, or those of the publisher, the editors and the reviewers. Any product that may be evaluated in this article, or claim that may be made by its manufacturer, is not guaranteed or endorsed by the publisher.
